# EDA and EDAR expression at different stages of hair follicle development in cashmere goats and effects on expression of related genes

**DOI:** 10.5194/aab-63-461-2020

**Published:** 2020-12-10

**Authors:** Zhihong Wu, Yu Wang, Wenjing Han, Kun Yang, Erhan Hai, Rong Ma, Zhengyang Di, Fangzheng Shang, Rui Su, Ruijun Wang, Zhiying Wang, Yanjun Zhang, Jinquan Li

**Affiliations:** 1 College of Animal Science, Inner Mongolia Agricultural University, Hohhot, Inner Mongolia Autonomous Region 010018, China; 2 College of Veterinary Medicine, Inner Mongolia Agricultural University, Hohhot, Inner Mongolia Autonomous Region 010018, China; 3 College of Chemistry and Life Science, Chifeng University, Chifeng, Inner Mongolia Autonomous Region 024000, China; 4 Key Laboratory of Animal Genetics, Breeding and Reproduction in Inner Mongolia Autonomous Region, Hohhot, Inner Mongolia Autonomous Region 010018, China; 5 Engineering Research Center for Goat Genetics and Breeding, Inner Mongolia Autonomous Region, Hohhot, Inner Mongolia Autonomous Region 010018, China; 6 Key Laboratory of Mutton Sheep Genetics and Breeding, Ministry of Agriculture, Hohhot, Inner Mongolia Autonomous Region 010018, China

## Abstract

This study is focused on the detection of ectodysplasin A (EDA) and ectodysplasin A receptor (EDAR) mRNA expression levels and protein positions in seven stages of cashmere goat fetus development (45, 55, 65, 75 95, 115, and 135 d), with the main goal of investigating the effect of EDA and EDAR on genes related to hair follicle development. Quantitative real-time polymerase chain reaction (RT-qPCR) was used to measure EDA and EDAR expression levels in seven stages of cashmere goat fetus development. Immunohistochemistry (IHC) was used to locate EDA and EDAR in the critical stage of fetal hair follicle development (45–135 d). EDA and EDAR expression in fetal fibroblasts and epithelial cells was interfered with by short hairpin RNA (sh-RNA). The results indicated that EDA and EDAR were both expressed in the skin tissue in the seven cashmere goat embryo stages. Moreover, EDA and EDAR play an important role in the formation of embryonic placode (Pc). After interfering with EDA and EDAR, the expression of BMP2, BMP4, noggin, β-catenin, TGF-β2, Wnt-10b, and NOTCH1 in fibroblasts and epithelial cells changed significantly. This study provides a theoretical and experimental basis for further studying the molecular regulation mechanism of hair follicle development.

## 1 Introduction

The Inner Mongolian cashmere goat is an important source of germplasm with high economic value. The growth and development of hair follicles have an important effect on the yield and quality of vellus. Cashmere goat hair follicles are divided into primary and secondary follicles. Primary hair follicles produce hair locks and secondary hair follicles produce cashmere. Primary hair follicles occur earlier with complete subsidiary structures, while secondary hair follicles occur later without sweat glands and hair shaft muscle. The main difference between the two is that primary hair follicles have sebaceous glands, while secondary hair follicles lack or have undeveloped sebaceous glands (Hardy, 1992; Paus and Cotsarelis, 1999). Early in the developmental process of the formation of the structure of cashmere goat hair follicles, we conducted a morphogenesis study and found that on gestational day 45, fetal epidermis was the only layer of flat cells that did not form primary follicles (Yin et al., 2005). Inner Mongolian cashmere goats develop the complete structure of their epidermis from gestational day 45 to gestational day 55; the primary follicles have not yet begun to develop during this period. Keratinocytes are aligned neatly on the epidermal base. During the development of the fetus from day 55 to day 65, the length of the hair buds increases significantly, and they penetrate into the cells of the dermis. From day 65 to day 75 of fetal development, the secondary follicle primordium can be observed. Most secondary follicles develop their complete structure by day 125 of fetal growth, and hair matrix cells surround the dermal papillae. Both dermal papillae and primary hair follicles have an oval shape, which facilitates the penetration of villi through the body surface. On day 135, some of the hair follicles are mature and the villi have pierced through the body surface (Zhang et al., 2006; Han et al., 2017). This whole process is not only affected by genetic, nutritional, and environmental factors, but also by complex physiological and biochemical processes.

Ectodysplasin A (EDA) is a unique signalling molecule in the tumor necrosis factor family (Kowalczyk-Quintas et al., 2014). EDA is a virulence gene for X-linked hypohidrotic ectodermal dysplasia (Kere et al., 1996). X-linked and autosomal forms of anhidrotic ectodermal dysplasia syndromes are characterized by deficient development of several ectodermal organs, including hair, teeth, and exocrine glands (Laurikkala et al., 2002; Wang et al., 2015). EDA-A1 (391 amino acids) and EDA-A2 (389 amino acids) are the longest and most common EDA transcripts, differing only by two amino acids (Srivastava et al., 1997). EDA-A1 and EDA-A2 receptors are named EDAR and XEDAR, respectively (Fujimoto et al., 2008). EDA-A2 overexpression did not cause a specific phenotype under the keratin 14 promoter in the ectoderm of transgenic mice. In contrast, overexpression of EDA-A1 resulted in alterations in a variety of ectodermal organs, most notably in extra organs (Mustonen et al., 2003). It is suggested that EDA-A1-EDAR plays a leading role in the morphogenesis and differentiation of skin-derived structures. EDA and its receptor EDAR are required for the normal development of some ectodermal appendages, including hair follicles (Fessing et al., 2006).

There is a complex relationship between a series of molecules that regulate the development of cashmere goat hair follicles (Schmidt-Ullrich and Paus, 2005; Ramos et al., 1995). Previous research conducted on EDA and EDAR has mainly focused on ectodermal dysplasia in humans and experimental animals (Garcin et al., 2016; Monreal et al., 1999). However, there has been a limited amount of such research relating to the cashmere goat. This study was based on a study of the occurrence and development of cashmere goat hair follicles. Quantitative real-time polymerase chain reaction (RT-qPCR) techniques were used to detect the expression of mRNA in cashmere goat skin tissue during seven developmental periods (45, 55, 65, 75, 95, 115, and 135 d). RNA interference was used to explore its effect on genes related to hair follicle development through interference with fetal fibroblasts and epithelial cell EDA and EDAR expression, providing a theoretical and experimental basis for exploring the molecular mechanism of hair follicle development.

## 2 Material and methods

### 2.1 Material

We selected a group of ewes that shared the same grazing and growth conditions at the Inner Mongolia Autonomous Region Hohhot Jinlai animal husbandry facility (Hohhot, China). Fetal skin samples were collected from cashmere goats at different gestational periods (45, 55, 65, 75, 95, 115, and 135 d; three goats for each stage), quickly placed in liquid nitrogen, frozen, and refrigerated at -80${}^{\circ }$ for later use. Effective anesthesia and aseptic operation were performed prior to the procedure. Diet adjustment should be done after operation. The fetal skin fibroblasts and epithelial cells of Inner Mongolian cashmere goats were preserved in the laboratory. All animal procedures were approved by the Inner Mongolia Agricultural University Animal Care and Use Committee (Hohhot, China) in accordance with the National Animal Care Standards.

### 2.2 Methods

#### 2.2.1 Detection of the gene expression of EDA and EDAR in the skin of Inner Mongolian cashmere goats using a RT-qPCR detecting system

Total RNA was extracted using the RNAiso Plus kit (Takara, China) according to the operating instructions and reverse-transcribed using an RNA reverse transcription kit. The relative expression of EDA and EDAR in goat skin tissue at seven fetal stages was detected using the real-time fluorescence quantitative method. The 20 µL reaction system volume contained 10 µL of 2X SuperReal PreMix, 1 µL of diluted reverse transcription product, 0.7 µL of each specific primer (10 µmol L${}^{-1}$), 0.4 µL of ROX (50x), and 7.2 µL of ddH2O. Reaction conditions were 95${}^{\circ }$ for 3 min, 95${}^{\circ }$ for 30 s, and 30 s at annealing temperature for 40 cycles. The Ct value was automatically generated using the default settings. Each sample was analyzed in three replicates.

The primer sequences of genes are shown in Table 1. Real-time quantitative polymerase chain reaction detecting system data were calculated using the 2${}^{-\Delta \Delta Ct}$ method. The experiment was repeated three times. Data were analyzed using SPSS 17.0 (IBM, USA) with a t test. Data were expressed as mean ± standard error or mean (SEM), and statistical significance was set at ${}^{*}P<0.05$ and ${}^{**}P<0.01$.

**Figure 1:**
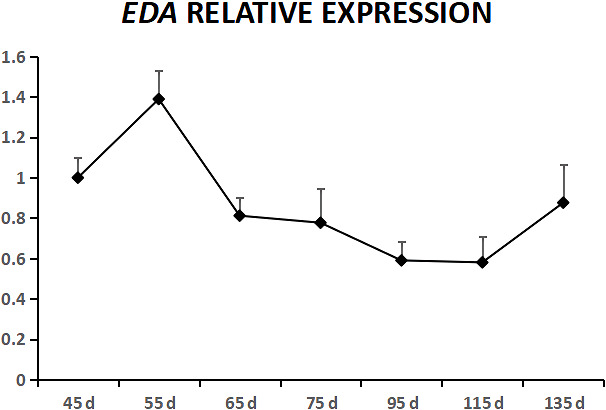
Relative expression levels of the EDA gene during the fetal period.

#### 2.2.2 Immunohistochemistry (IHC) to locate EDA and EDAR in the fetal hair follicles of cashmere goats after 45, 55, 65, 75, 95, 115, and 135 d

Skin samples were obtained from three different cashmere goats at different hair follicle development stages. Skin biopsy sections were fixed with paraformaldehyde for 24 h. The samples were then dehydrated with 70 %, 80 %, and 95 % alcohol, respectively, for 1 h. Next, xylene was used twice for approximately 1 h each time until the organ was transparent through light. Samples were then soaked with a soft wax and xylene mixture for 2 h, before finally being soaked with geocerain in an incubator (65–70${}^{\circ }$) overnight. The samples were cut into paraffin sections (5 µm thick) using a microtome and dried at 70${}^{\circ }$. They were then dewaxed and backwashed with xylene and alcohol, respectively, before washing with phosphate-buffered saline three times for antigen repair. The IHC kits (Kit-9710) and 3, 3${}^{\prime }$-diaminobenzidine kit (DAB-0031) were purchased from Xinmai Co., Ltd. (Fuzhou, China). IHC experimental protocols were performed according to the kit manual. Antigens for EDA and EDAR were purchased from Abcam Inc. (England), and the product numbers are ab203075 and ab198168. Slides were mounted with neutral balsam (Xinmai Co., Ltd., Fuzhou, China) and photographed using a microscope (Nikon 80i) connected to a digital camera system.

**Figure 2:**
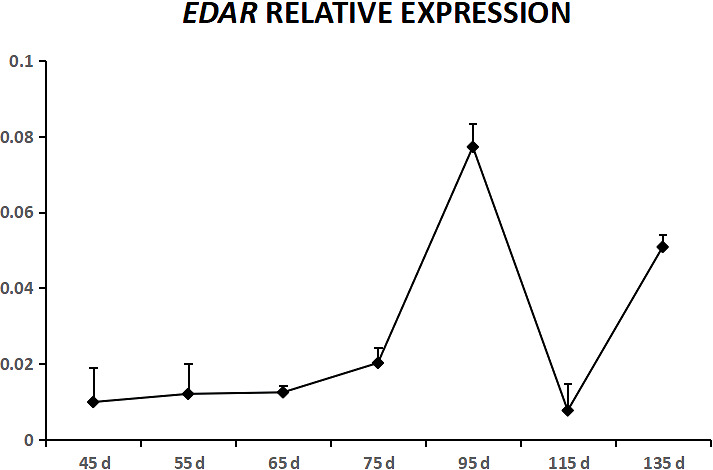
Relative expression levels of the EDAR gene during the fetal period.

#### 2.2.3 Effects of EDA and EDAR interference on related gene expression

The fetal skin fibroblasts and epithelial cells of Inner Mongolian cashmere goats were preserved in the laboratory. The carrier was designed and synthesized by Bioengineering Co., Ltd. (Shanghai).

#### 2.2.4 Transfection of fetal skin fibroblasts and epithelial cells from Inner Mongolian cashmere goats using an interference vector

Prior to transfection, cashmere goat fibroblasts and epithelial cells were seeded at a density of 4–5 × 105 on 24-well plates. Dulbecco's modified eagle medium (0.5 mL) containing 10 % fetal bovine serum was added. The cells were grown to 70 % confluence. Lipofectamine^®^ LTX Reagent (2 µL; Invitrogen) was added to 50 µL Opti-MEM (Gibco) and mixed gently. DNA (0.8 µg) was added to 50 µL Opti-MEM (Gibio) and mixed gently with 0.8 µL PLUS (Invitrogen). After incubating for 5 min at room temperature, the two solutions were mixed and gently added to the cells. The cell culture plate was shaken back and forth and placed in a 5 % CO${}_{2}$ incubator for 4–6 h at 37${}^{\circ }$. The complex was then removed, the medium replaced with serum, and fluorescence observed after 48 h.

#### 2.2.5 RT-qPCR analysis of related genes in fibroblasts and epithelial cells by EDA and EDAR interference

In this study, β-actin was used as an internal control. Transfected short hairpin EDA (sh-EDA) and short hairpin EDAR (sh-EDAR) cells were used as the experimental group and untransfected cells as a control group. BMP2, BMP4, noggin, Wnt-10b, and NOTCH1 transcription level changes were detected using RT-qPCR. Experimental and analytical methods are the same as described in Sect. 2. The primer sequences of the above genes are shown in Table 1.

**Table 1:** The mRNA primer sequence.

Gene name		Sequence	Product (bp)
			
β-actin	Forward Reverse	5${}^{\prime }$-GGCAGGTCATCACCATCGG-3${}^{\prime }$ 5${}^{\prime }$-CGTGTTGGCGTAGAGGTCTTT-3${}^{\prime }$	158
EDA	Forward Reverse	5${}^{\prime }$-GACCTTCTGGTGCTGCTGA-3${}^{\prime }$ 5${}^{\prime }$-ACTTGAATCGCTGACCCTTG-3${}^{\prime }$	88
EDAR	Forward Reverse	5${}^{\prime }$-GTAGACGTGAGGTTGCACTGG-3${}^{\prime }$ 5${}^{\prime }$-CTGGTGTTTGCTGGGTGGT-3${}^{\prime }$	114
BMP2	Forward Reverse	5${}^{\prime }$-CCTTTATATGTGGACTTCAGTG-3${}^{\prime }$ 5${}^{\prime }$-GCCTTGGGAATCTTAGAGTTA-3${}^{\prime }$	179
BMP4	Forward Reverse	5${}^{\prime }$-GCGAGCCATGCTAGTTTGATACC-3${}^{\prime }$ 5${}^{\prime }$-GTGGAAGCTCCTCACGGTGTTG-3${}^{\prime }$	306
Noggin	Forward Reverse	5${}^{\prime }$-GGCCAGCACTATCTCCACAT-3${}^{\prime }$ 5${}^{\prime }$-GCGTCTCGTTCAGATCCTTC-3${}^{\prime }$	115
β-catenin	Forward Reverse	5${}^{\prime }$-GACCACAAGCAGAGTGCT-3${}^{\prime }$ 5${}^{\prime }$-TGTCAGGTGAAGTCCTAAA-3${}^{\prime }$	100
TGF-β2	Forward Reverse	5${}^{\prime }$-AAAACCAGAGCAGAGGGTGAATG-3${}^{\prime }$ 5${}^{\prime }$-AAGGTGCAGCAGGGACAGTGTAA-3${}^{\prime }$	122
Wnt-10b	Forward Reverse	5${}^{\prime }$-ATGGACTTCGGGGAGAAGTT-3${}^{\prime }$ 5${}^{\prime }$-CTTGCATTTCCGCTTCAAGT-3${}^{\prime }$	138
NOTCH1	Forward Reverse	5${}^{\prime }$-ATGTATGTGGCTGTGGTGG-3${}^{\prime }$ 5${}^{\prime }$-CGCCGCTTCTTCTTGCTG-3${}^{\prime }$	143

## 3 Results

### 3.1 mRNA expression of EDA and EDAR in fetal skin tissue of Inner Mongolian cashmere goats

EDA was expressed in cashmere goat fetus skin tissue during all seven experimental time points (Fig. 1). From 45 to 55 d, EDA expression increased and reached the highest value; from 55 to 115 d, EDA expression decreased, and from 115 to 135 d, EDA expression increased. EDAR was expressed in cashmere goat fetus skin tissue during all 10 experimental time points (Fig. 2). From 45 to 95 d, the expression of EDAR increased and reached the maximum value at 95 d; from 95 to 115 d, the expression of EDAR decreased, and from 115 to 135 d, the expression of EDAR increased.

### 3.2 Localization of EDA and EDAR during the seven stages

**Figure 3:**
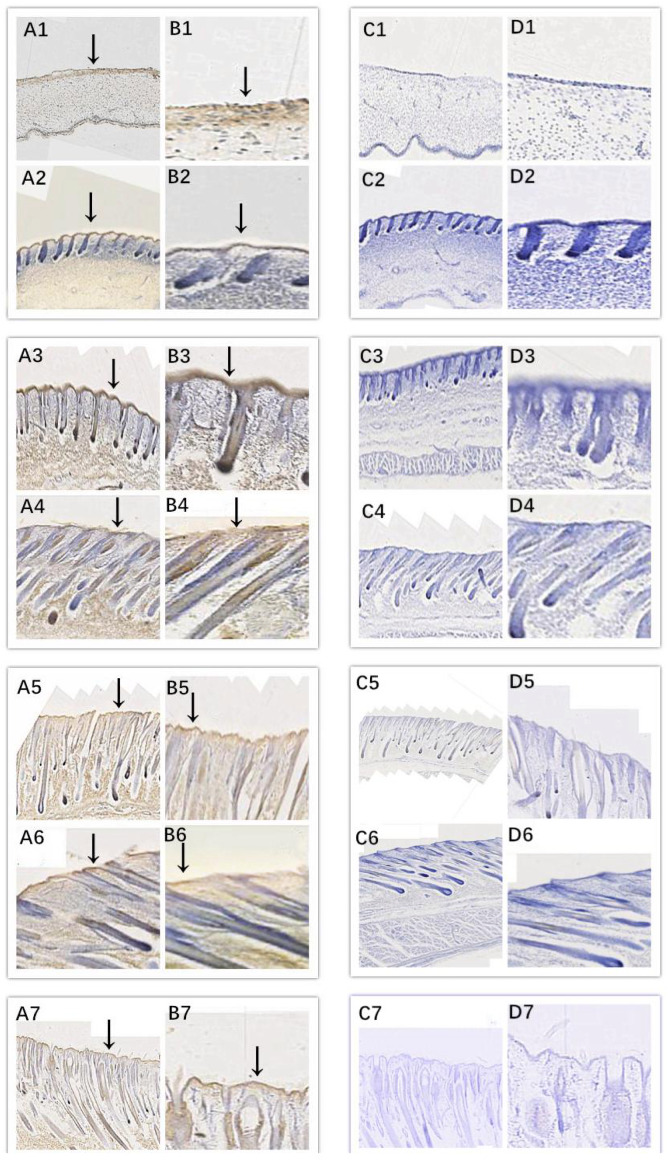
Location of EDA expression. Note: A1–A7, B1–B7, C1–C7, and D1–D7 are longitudinal section views of the skin tissue of Inner Mongolian cashmere goats at 45, 55, 65, 75, 95, 115, and 135 d, respectively. The magnification of the results in the first and third columns was × 100, that of the second and fourth columns was × 400, and the third and fourth columns were negative controls. A1–A7, B1–B7, C1–C7, and D1–D7 show the follicle slitting results.

**Figure 4:**
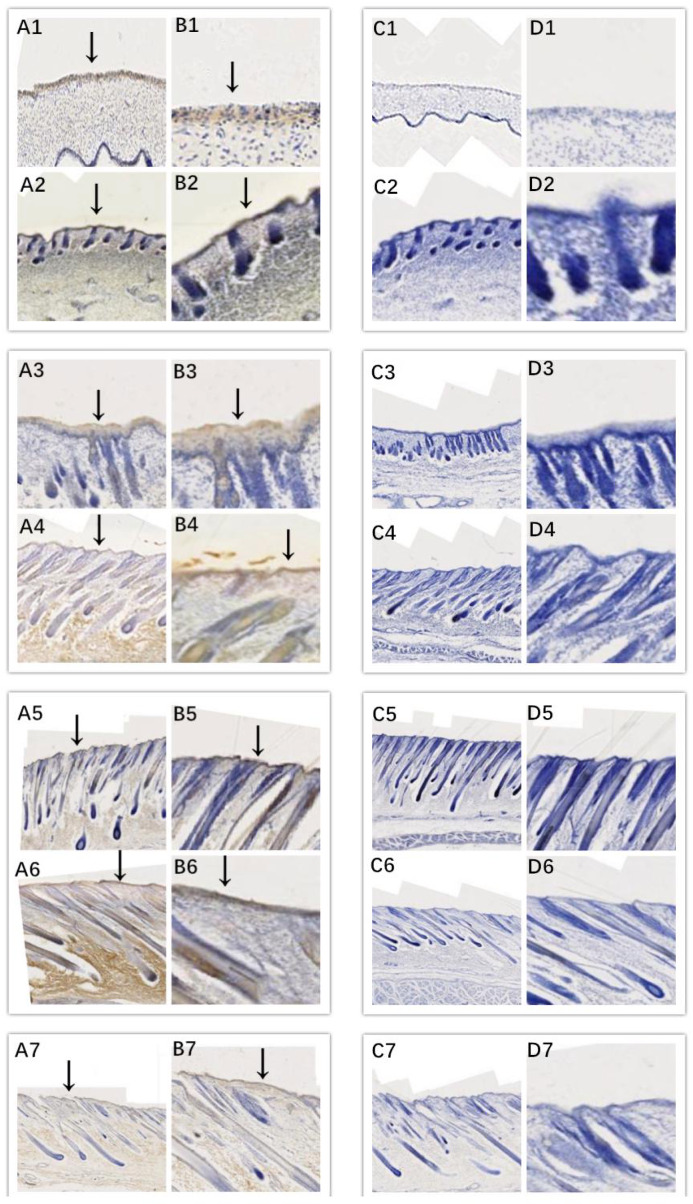
Location of EDAR expression. Note: A1–A7, B1–B7, C1–C7, and D1–D7 are longitudinal section views of the skin tissue of Inner Mongolian cashmere goats at 45, 55, 65, 75, 95, 115, and 135 d, respectively. The magnification of the results in the first and third columns was × 100, that of the second and fourth columns was × 400, and the third and fourth columns were negative controls. A1–A7, B1–B7, C1–C7, and D1–D7 show the follicle slitting results.

In this study, we successfully identified the expression sites of EDA and EDAR in the skin of cashmere goats at different stages. The results showed that EDA (Fig. 3) and EDAR (Fig. 4) were mainly expressed in the placode (Pc). The above results show that EDA and EDAR play an important role in hair follicle development in cashmere goats.

### 3.3 Effects of EDA and EDAR interference on related gene expression

#### 3.3.1 Identification of interfering carrier by enzyme digestion

**Figure 5:**
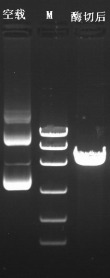
Identification of the pSGU6/GFP/Neo sh-RNA vector. Note: left of the marker is an empty PCR band of 413 bp, and on the right is a positive PCR fragment of the 429 bp vector.

The plasmid of the interference carrier pSGU6/GFP/Neo sh-RNA was digested with Bbs(?) and Hind(?), resulting in a gene fragment of 429 bp (Fig. 5), indicating that the interference carrier pSGU6/GFP/Neo sh-RNA had been constructed successfully.

#### 3.3.2 Transfection of cells with EDA and EDAR interference vector

**Figure 6:**
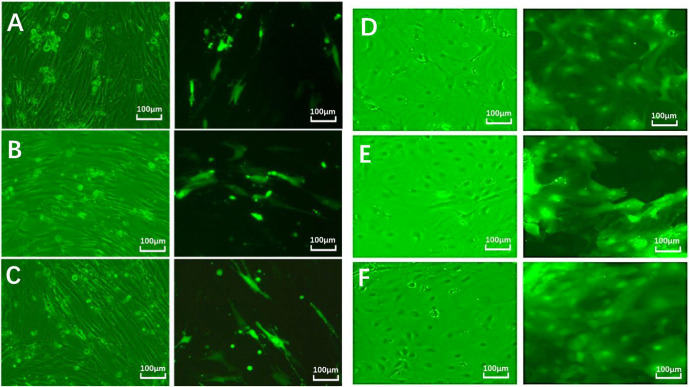
Cell transfection. Note: green fluorescent expression after transfection of fibroblasts (left) and epithelial cells (right) for 24 h (100 µm). Note: A–D show a cell microscope diagram after transfection of sh-EDA for 24 h under bright-field (left) and fluorescence conditions (right). B–E show a cell microscope diagram after transfection of sh-EDAR for 24 h under bright-field (left) and fluorescence conditions (right). C–F show the negative control vector (NC-sh-RNA).

After 24 h, fibroblast and epithelial cell (Fig. 6) transfection was observed under phase contrast and fluorescence microscopy. Based on cell alignment, it was shown that the interference vector and negative control vector were successfully transferred into goat fetal skin fibroblasts and epithelial cells. After transfection, the vector was green under fluorescent microscopy and could therefore be used for subsequent experiments.

#### 3.3.3 Effects of EDA and EDAR interference on the related gene expression in fibroblasts

**Figure 7:**
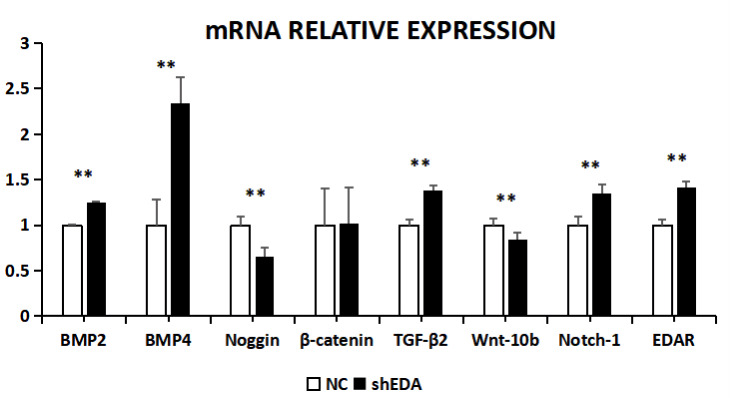
The mRNA relative expression of BMP2, BMP4, noggin, β-catenin, TGF-β2, Wnt-10b, NOTCH1, and EDAR after EDA interference Note: ${}^{*}$ significant difference. ${}^{**}$ Extremely significant difference.

The expression of BMP2, BMP4, noggin, β-catenin, TGF-β2, Wnt-10b, NOTCH1, and EDAR at the transcriptional level was 1.2499, 2.3403, 0.6545, 1.0163, 1.3760, 0.8397, 1.3519, and 1.416 times higher, respectively, than that of the control group after EDA interference by RT-qPCR (Fig. 7). The relative expression of noggin and Wnt-10b decreased, while the relative expression of BMP2, BMP4, β-catenin, TGF-β2, NOTCH1, and EDAR increased. Compared with the control groups, the expression of BMP2, BMP4, noggin, TGF-β2, Wnt-10b, NOTCH1, and EDAR at the transcription level exhibited significant differences (P<0.01), while the expression of β-catenin showed no significant difference (P>0.05).

**Figure 8:**
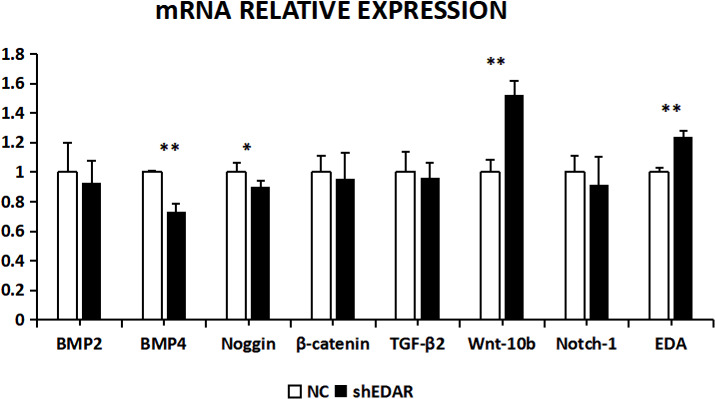
The mRNA relative expression of BMP2, BMP4, noggin, β-catenin, TGF-β2, Wnt-10b, NOTCH1, and EDA after EDAR interference. Note: ${}^{*}$ significant difference. ${}^{**}$ Extremely significant difference

After EDAR interference, the expression of BMP2, BMP4, noggin, β-catenin, TGF-β2, Wnt-10b, NOTCH1, and EDA at the transcriptional level was 0.9288, 0.7355, 0.8992, 0.9528, 0.9616, 1.5195, 0.9119, and 1.24 times higher, respectively, than that of the control group (Fig. 8). In addition to Wnt-10b and EDA, the relative expression of the other six was reduced. Compared with the control groups, the expression of BMP4, Wnt-10b, and EDA at the transcription level exhibited significant differences (P<0.01). The expression of noggin was also different (P<0.05). The expression of BMP2, β-catenin, TGF-β2, and NOTCH1 showed no significant difference (P>0.05).

#### 3.3.4 Effects of EDA and EDAR interference on the related gene expression in epithelial cells

**Figure 9:**
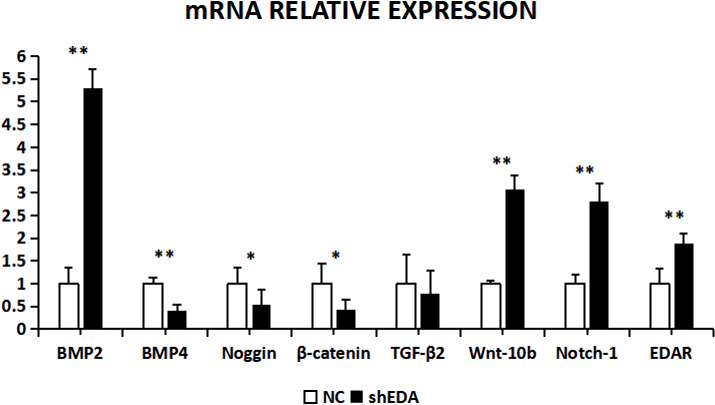
The mRNA relative expression of BMP2, BMP4, noggin, β-catenin, TGF-β2, Wnt-10b, NOTCH1, and EDAR after EDA interference. Note: ${}^{*}$ significant difference. ${}^{**}$ Extremely significant difference.

The expression of BMP2, BMP4, noggin, β-catenin, TGF-β2, Wnt-10b, NOTCH1, and EDAR at the transcriptional level was 5.2873, 0.4067, 0.5317, 0.4261, 0.7835, 3.0798, 2.8041, and 1.89 times higher, respectively, than that of the control group after EDA interference, as observed using RT-qPCR (Fig. 9). The relative expression of BMP4, β-catenin, noggin, and TGF-β2 decreased, whereas that of BMP2, Wnt10b, NOTCH1, and EDAR increased. The transcriptional expression of BMP2, BMP4, Wnt-10b, NOTCH1, and EDAR significantly differed between the cells with and without EDA interference (P<0.01), whereas the expression of TGF-β2 did not differ (P>0.05).

**Figure 10:**
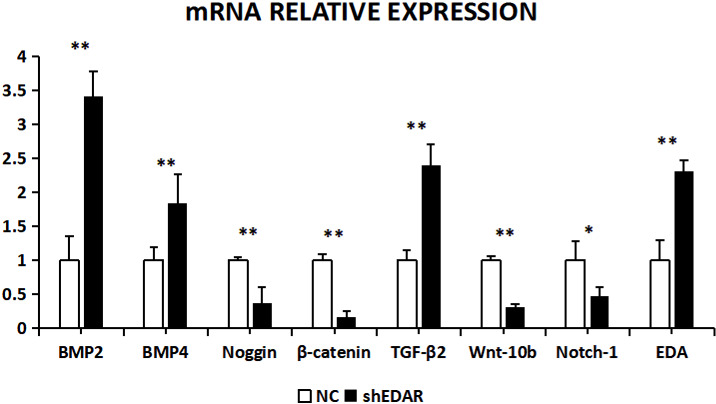
The mRNA relative expression of BMP2, BMP4, noggin, β-catenin, TGF-β2, Wnt-10b, NOTCH1, and EDA after EDAR interference. Note: ${}^{*}$ significant difference. ${}^{**}$ Extremely significant difference

With EDAR interference, the transcriptional expression of BMP2, BMP4, noggin, β-catenin, TGF-β2, Wnt-10b, NOTCH1, and EDA was 3.4183, 1.8446, 0.3742, 0.1619, 2.401, 0.3216, 0.473, and 2.31 times higher, respectively, than that of the controls (Fig. 10). The expression of BMP2, BMP4, noggin, Wnt-10b, TGF-β2, NOTCH1, and EDA was significantly lower in the cells with EDAR interference than in the controls (P<0.01). The expression of NOTCH1 also differed between the experimental and control groups (P<0.05).

## 4 Discussion

The development of hair follicles is induced by the interaction between epithelial cells and fibroblasts. From the beginning of epithelial cells forming the Pc, to fibroblast subsidence to form dermal condensate, and finally to the development of hair follicles, this process is active in a large number of signalling pathways, such as the Wnt/β-catenin signalling pathway, TGF-β signalling pathway, and mitogen-activated protein kinase (MAPK) signalling pathway. In previous studies, it was discovered that the cell fates of epithelial cells and fibroblasts are different during the development of hair follicles. The fundamental reason may be that these key signalling molecules play different roles in the two cells. In this study, we found that EDA and EDAR were expressed in the Pc during the seven fetal stages of Inner Mongolian cashmere goats. Although the expression trends for EDA and EDAR were inconsistent, they were located on the Pc, which indicated that their roles in the Pc might be different, and subsequent sh-RNA corroborates this.

IHC results showed that EDA and EDAR were mainly expressed in the Pc during the seven fetal stages of Inner Mongolian cashmere goats, which indicated that they played an important role in the formation of Pc and the formation of hair follicles by basal subsidence. When combined with the RT-qPCR results, EDA was highly expressed at 45 and 55 d; at this stage, the Pc began to form and the dermis began to clearly subside. This indicates that the main role of EDA in the development of fetal skin hair follicles in Inner Mongolian cashmere goats is the development of Pc and the induction of hair follicles. Different from EDA, EDAR displayed low expression at 45 and 55 d and only high expression at 95 and 135 d. Therefore, we speculate that EDAR may be indirectly involved in the formation of Pc, but its main role is to promote the rapid differentiation of hair follicles and maintain structural stability during the hair follicle maturation stage. In the cell sh-RNA test, it was found that the expression of EDA and EDAR in epithelial cells or fibroblasts increases the expression of the other, which indicated that they may have a complementary relationship, also confirming that the stable expression of EDA and EDAR is essential for ectoderm development (Iida et al., 2014; Zeng et al., 2016).

Bone morphogenetic proteins (BMPs) are a family of secretory signalling molecules with multiple functions in which BMP2 and BMP4 are the key genes. They play a key role in the layering of mammalian epidermis (Zhu et al., 2014) and the regulation of hair regeneration in mouse skin (Plikus et al., 2008). Noggin is a BMP inhibitor during the development of mouse teeth. Noggin can inhibit conduction of the BMP signalling pathway and the differentiation of ameloblasts (Cao et al., 2013). Inhibiting noggin can promote the expression of BMP2 and ultimately stimulate fat-derived stem-cell osteogenesis (Fan et al., 2013). We found through cell sh-RNA that the expression of noggin in epithelial cell sh-EDA decreased sharply and the expression of BMP2 increased sharply, but the expression of BMP4 decreased. This may be because EDAR possibly inhibits both noggin and BMP4 in epithelial cells, which indirectly led to a sharp increase in BMP2 expression. However, the fibroblast sh-EDAR results showed that there was no significant change in BMP2, and the mechanism required further study. We found that the decrease in noggin expression increased the expression of BMP2 and BMP4 in epithelial cell sh-EDAR and fibroblast sh-EDA, which indicates that the effect of EDA in epithelial cells and EDAR in fibroblasts may be because noggin has similar effects, and both are inhibitors of noggin.

The Wnt/β-catenin signalling pathway is generally divided into the classical Wnt/β-catenin signalling pathway and nonclassical Wnt/β-catenin signalling pathway; the most obvious difference between these two branches is whether or not β-catenin is involved. In general, the role of β-catenin in the classic Wnt/β-catenin signalling pathway is very important, while the nonclassical Wnt/β-catenin signalling pathway does not require β-catenin to participate (Axelrod et al., 1998). EDA and EDAR are generally considered to be activators of the Wnt/β-catenin signalling pathway, but EDA and EDAR activation is not necessary during the development of skin hair follicles as they only play a role in the development and maintenance of primary hair follicles (Zhang et al., 2009). In the fibroblast sh-RNA test, we can see that there is no significant change in the expression of β-catenin in both sh-EDA and sh-EDAR, but there is a significant difference in Wnt-10b. We speculate that in fibroblasts, EDA and EDAR do not participate in signal transduction of the classic Wnt/β-catenin signalling pathway but regulate the nonclassical Wnt/β-catenin signalling pathway, while in epithelial cells, EDA and EDAR may be involved in regulation of the classical Wnt/β-catenin signalling pathway. In addition to the effects of EDA and EDAR on classical and nonclassical Wnt/β-catenin signalling pathways, they are also indirectly involved in the regulation of TGF-β2 (Działo et al., 2018) and NOTCH1 (Collu et al., 2014).

Hair follicle development involves many signalling pathways. It has been reported that EDA and EDAR play a role in mouse hair follicle formation and growth. However, changes in hair follicles are complex physiological and biochemical processes involving multiple closely related genes. Therefore, the mechanism through which EDA and EDAR affect hair follicle growth requires further exploration. The study of these genes in relation to the development of hair follicles in goats is particularly in need of additional study.

## Data Availability

All data produced and used during the study appear in the paper.

## References

[bib1.bib1] Axelrod JD, Miller JR, Shulman JM, Moon RT, Perrimon N (1998). Differential recruitment of Dishevelled provides signaling specificity in the planar cell polarity and Wingless signaling pathways. Genes Dev.

[bib1.bib2] Cao H, Jheon A, Li X, Sun Z, Wang J, Florez S, Zhang Z, McManus MT, Klein OD, Amendt BA (2013). The Pitx2:miR-200c/141:noggin pathway regulates Bmp signaling and ameloblast differentiation. Development.

[bib1.bib3] Collu GM, Hidalgo-Sastre A, Brennan K (2014). Wnt-Notch signalling crosstalk in development and disease. Cell Mol Life Sci.

[bib1.bib4] Działo E, Tkacz K, Błyszczuk P (2018). Crosstalk between the TGF-β and WNT signalling pathways during cardiac fibrogenesis. Acta Biochim Pol.

[bib1.bib5] Fan J, Park H, Tan S, Lee M (2013). Enhanced osteogenesis of adipose derived stem cells with Noggin suppression and delivery of BMP-2. PLoS ONE.

[bib1.bib6] Fessing MY, Sharova TY, Sharov AA, Atoyan R, Botchkarev VA (2006). Involvement of the Edar Signaling in the Control of Hair Follicle Involution (Catagen). Am J Pathol.

[bib1.bib7] Fujimoto A, Kimura R, Ohashi J, Omi K, Yuliwulandari R, Batubara L, Mustofa MS, Samakkarn U, Settheetham-Ishida W, Ishida T, Morishita Y, Furusawa T, Nakazawa M, Ohtsuka R, Tokunaga K (2008). A scan for genetic determinants of human hair morphology: EDAR is associated with Asian hair thickness. Hum Mol Genet.

[bib1.bib8] Garcin CL, Huttner KM, Kirby N, Schneider P, Hardman MJ (2016). Ectodysplasin A pathway contributes to human and murine skin repair. J Invest Dermatol.

[bib1.bib9] Han W, Li X, Wang L, Wang H, Yang K, Wang Z, Wang R, Su R, Liu Z, Zhao Y, Zhang Y, Li J (2017). Expression of Fox-related genes in the skin follicles of Inner Mongolia Cashmere Goat. Asian-australas J Anim Sci.

[bib1.bib10] Hardy MH (1992). The secret life of the hair follicle. Trends in Genetics.

[bib1.bib11] Iida Y, Hibiya K, Inohaya K, Kudo A (2014). Eda/Edar signaling guides fin ray formation with preceding osteoblast differentiation, as revealed by analyses of the medaka all-fin less mutant afl. Dev Dyn.

[bib1.bib12] Kere J, Srivastava AK, Montonen O, Zonana J, Thomas N, Ferguson B, Munoz F, Morgan D, Clarke A, Baybayan P, Chen EY, Ezer S, Saarialho-Kere U, de la Chapelle A, Schlessinger D (1996). X-linked anhidrotic (hypohidrotic) ectodermal dysplasia is caused by mutation in a novel transmembrane protein. Nature Genetics.

[bib1.bib13] Kowalczyk-Quintas C, Willen L, Dang A T, Sarrasin H, Tardivel A, Hermes K, Schneider H, Gaide O, Donzé O, Kirby N, Headon DJ, Schneider P (2014). Generation and characterization of function-blocking anti-ectodysplasin A (EDA) monoclonal antibodies that induce ectodermal dysplasia. J Biol Chem.

[bib1.bib14] Laurikkala J, Pispa J, Jung HS, Nieminen P, Mikkola M, Wang X, Saarialho-Kere U, Galceran J, Grosschedl R, Thesleff I (2002). Regulation of hair follicle development by the TNF signal ectodysplasin and its receptor Edar. Development.

[bib1.bib15] Monreal AW, Ferguson BM, Headon DJ, Street SL, Overbeek PA, Zonana J (1999). Mutations in the human homologue of mouse dl cause autosomal recessiveand dominant hypohidrotic ectodermal dysplasia. Nat Genet.

[bib1.bib16] Mustonen T, Pispa J, Mikkola ML, Pummila M, Kangas AT, Pakkasjärvi L, Jaatinen R, Thesleff I (2003). Stimulation of ectodermal organ development by Ectodysplasin-A1. Dev Biol.

[bib1.bib17] Paus R, Cotsarelis G (1999). The biology of hair follicles. N Engl J Med.

[bib1.bib18] Plikus MV, Mayer JA, de la Cruz D, Baker RE, Maini PK, Maxson R, amd Chuong CM (2008). Cyclic dermal BMP signalling regulates stem cell activation during hair regeneration. Nature.

[bib1.bib19] Ramos V, Giebink DL, Fisher JG, Christensen LC (1995). Complete dentures for a child with hypohidrotic ectodermal dysplasia: a clinical report. J Prosthet Dent.

[bib1.bib20] Schmidt-Ullrich R, Paus R (2005). Molecular principles of hair follicle induction and morphogenesis. BioEssays.

[bib1.bib21] Srivastava AK, Pispa J, Hartung AJ, Du Y, Ezer S, Jenks T, Shimada T, Pekkanen M, Mikkola ML, Ko MS, Thesleff I, Kere J, Schlessinger D (1997). The Tabby Phenotype is Caused by Mutation in a Mouse Homologue of the EDA Gene that Reveals Novel Mouse and Human Exons and Encodes a Protein (Ectodysplasin-A) with Collagenous Domains. P Natl Acad Sci USA.

[bib1.bib22] Wang XJ, Jian-Ning HE, Liu N (2015). Research Progress on EDA Gene and its Role in the Development of Animal Hair Follicle. China Animal Husbandry & Veterinary Medicine.

[bib1.bib23] Yin J, Li J, Zhang Y, Li C, Guo Z (2005). Study on gene expression of skin from adult Inner Mongolia Cashmere Goat. Chinese Journal of Animal and Veterinary Sciences.

[bib1.bib24] Zeng B, Xiao X, Li S, Lu H, Lu J, Zhu L, Yu D, Zhao W (2016). Eight Mutations of Three Genes (EDA, EDAR, and WNT10A) Identified in Seven Hypohidrotic Ectodermal Dysplasia Patients. Genes (Basel).

[bib1.bib25] Zhang Y, Tomann P, Andl T, Gallant NM, Huelsken J, Jerchow B, Birchmeier W, Paus R, Piccolo S, Mikkola ML, Morrisey EE, Overbeek PA, Scheidereit C, Millar SE, Schmidt-Ullrich R (2009). Reciprocal requirements for EDA/EDAR/NF-kappaB and Wnt/beta-catenin signaling pathways in hair follicle induction. Dev Cell.

[bib1.bib26] Zhang Y, Yin J, Li C, Li J (2006). Study on development of skin and hair follicle from fetal Inner Mongolian Arbas Cashmere Goats. Acta Veterinaria et Zootechnica Sinica.

[bib1.bib27] Zhu X, Liu Y, Dai Z, Zhang X, Yang X, Li Y, Qiu M, Fu J, Hsu W, Chen Y, Zhang Z (2014). BMP-FGF signaling axis mediates Wnt-induced epidermal stratification in developing mammalian skin. PLoS Genet.

